# A New Highlight of *Ephedra alata* Decne Properties as Potential Adjuvant in Combination with Cisplatin to Induce Cell Death of 4T1 Breast Cancer Cells In Vitro and In Vivo

**DOI:** 10.3390/cells9020362

**Published:** 2020-02-04

**Authors:** Fairouz Sioud, Souheila Amor, Imène ben Toumia, Aida Lahmar, Virginie Aires, Leila Chekir-Ghedira, Dominique Delmas

**Affiliations:** 1 Research Unit Bioactive Natural Products and Biotechnology UR17ES49, Faculty of Dental Medicine of Monastir, University of Monastir, Avicenne street, Monastir 5000, Tunisia; fairouz.sioud@yahoo.fr (F.S.); ben.toumia.imene@gmail.com (I.b.T.); elahmar.aida@gmail.com (A.L.); leila.chekir@laposte.net (L.C.-G.); 2 Université de Bourgogne Franche-Comté, F-21000 Dijon, France; Souheila.Amor@u-bourgogne.fr (S.A.); virginie.aires02@u-bourgogne.fr (V.A.); 3 INSERM Research Center U1231—Cancer and Adaptive Immune Response Team, F-21000 Dijon, France; 4 Centre anticancéreux Georges François Leclerc Center, F-21000 Dijon, France

**Keywords:** *Ephedra*, cisplatin, polyphenols, breast cancer, chemosensibilisation

## Abstract

Despite major advances in the last 10 years, whether in terms of prevention or treatment, the 5 year survival rate remains relatively low for a large number of cancers. These therapeutic failures can be the consequence of several factors associated with the cellular modifications or with the host by itself, especially for some anticancer drugs such as cisplatin, which induces a nephrotoxicity. In the strategy of research for active molecules capable both of exerting a protective action against the deleterious effects of cisplatin and exerting a chemosensitizing action with regard to cancer cells, we tested the potential effects of *Ephedra alata* Decne extract (E.A.) rich in polyphenolic compounds towards a 4T1 breast cancer model in vitro and in vivo. We showed that E.A. extract inhibited cell viability of 4T1 breast cancer cells and induced apoptosis in a caspase-dependent manner, which involved intrinsic pathways. Very interestingly, we observed a synergic antiproliferative and pro-apoptotic action with cisplatin. These events were associated with a strong decrease of breast tumor growth in mice treated with an E.A./cisplatin combination and simultaneously with a decrease of hepato- and nephrotoxicities of cisplatin.

## 1. Introduction

Cancer is infamous as the leading cause of death in the world. Breast cancer is the second leading cause of cancer-related deaths among women worldwide, and accounts for approximately 25% of total cancer cases and 15% of total cancer-related deaths [1]. To treat this type of cancer, there are several active modalities available, such as surgery, hormone therapy, cytotoxic therapy, radiation therapy, or a combination of these treatments [2]. However, the restricted region for the surgical procedure, the development of drug-resistance, and patients who suffer from side effects are the most important factors that limit the total success of these treatment modalities [3,4]. Indeed, for example, cisplatin (CDDP), which is a highly effective chemotherapeutic agent for a variety of cancers, including breast cancer, presents more side effects, including genotoxicity, nephrotoxicity, and acute myelotoxicity [5,6], and subsequently limits its use. Nevertheless, despite this toxicity, the use of cisplatin like taxol remain, for the moment, the only bulwark against very aggressive cancers such as breast cancer, in particular that which is diagnosed as triple negative. Indeed, triple negative breast cancers (TNBCs) represent a challenge due to the heterogeneity of the disease, but also due to the absence of well-defined molecular targets [7,8], and some combinations of compounds with platinum salts could be more efficient against TNBCs [9]. In this way, for several years, numerous studies have focused on finding molecules capable of reducing the side effects of cytotoxic agents on normal cells by modulating, for example, cytochrome P450 activity, phase II enzyme activity, or by increasing the effectiveness of chemotherapeutic drugs. This increase in chemosensitivity is important in clinical practice because the toxicity of the drugs is often a factor limiting the use of these anticancer agents. In this way, we and others have thus shown that the use of natural molecules such as polyphenols can modulate the metabolism enzymes of xenobiotics, but also modulate the pathways leading to the death of cancer cells that make it possible to restore sensitization to anticancer agents. For example, resveratrol, curcumin, vitamin E, or gallic acid can protect cells from cisplatin-induced genotoxicity [10,11,12]. Very interestingly, among these natural compounds, some of them can, at the same time, synergize with various anticancer drugs. Indeed, we have shown that resveratrol, a polyphenol of grapevine, and its metabolites were able to sensitize various colon cancer cells to anticancer drugs, including 5-fluoro-uracil, oxaliplatin, or 7-ethyl-10-hydroxycamptothecin (SN38), through a disruption of cell cycle, an induction of DNA damages, and/or apoptotic process [13,14]. This emblematic polyphenol is not the only one that can act in synergy with anticancer drugs—curcumin and echistatin have also previously been shown to enhance cisplatin sensitivity by modulating signaling pathways in cancer cells [15,16].

In this strategy of research for active molecules capable both of exerting a protective action against the deleterious effects of cisplatin with respect to normal cells, but also of simultaneously exerting a chemosensitizing action with regard to cancer cells, we have recently focused on the potential use of a medicinal plant belonging the *Ephedraceae* family, *Ephedra alata* Decne, which has been described as decreasing the side effects of chemotherapy [17,18]. Primary species of *Ephedra* are represented by *Ephedra sinica*, which has been used in China for more than 5000 years for the treatment of cold, bronchial asthma, cough, fever, flu, headache, edema, and allergies [19]. The major active ingredients of *Ephedra* are ephedrine alkaloids. The high content of ephedrine alkaloids limits the use of these plants due to potential health risk such as adverse cardiovascular and cerebrovascular events that are possibly associated with the use of dietary supplement preparations containing E-type alkaloids. Subsequently, the Food and Drug Administration (FDA) banned all over the counter drugs containing ephedrine. However, a recent study highlights that among *Ephedraceae* family, which includes approximately 67 species, mainly in the desert areas of Asia, America, Europe, and North Africa, various *Ephedra* species do not present the same total alkaloid content (TAC), total phenolic content (TPC), and total flavonoid content (TFC) [20]. In particular, the authors showed that *Ephedra alata* Decne presents a high content of flavonoids and phenolic compounds (53.3 ± 0.1 mg gallic acid equivalents per gram dry weight, 2.8 mg quercetin equivalents per gram dry weight, respectively). However, the qualitative and quantitative content of *Ephedra alata* Decne is also dependent of the plant origin. Therefore, it appears important to determine the presence or absence of ephedrine, which could lead to toxicological effects and the level of flavonoids, tannins, and miscellaneous compounds [21] that have been described with antioxidant [22], anti-inflammatory [23,24], hepatoprotective [25,26], antibacterial [27,28], and anticancer activities [29,30].

In the present study, we analyzed the specific content of *Ephedra alata* Decne, which was harvested from the Sahara of Tataouine, a region situated in southeast of Tunisia, and its potential antitumoral effect on breast cancer progression. We highlighted a high content of polyphenolic compounds, especially of quercetin and derivatives without the presence of ephedrine or pseudoephedrine. The extract obtained indicated an antiproliferative activity against 4T1 murine mammary carcinoma cells and, very interestingly, a synergic antiproliferative action with cisplatin. These actions involved the induction of the proteolytic caspase pathway through a mitochondrial activation. Moreover, these events were found to be associated with a decrease of breast tumor growth in mice treated with an *Ephedra alata* Decne extract (E.A.)/cisplatin combination.

## 2. Materials and Methods

### 2.1. Cell Lines

Murine mammary carcinoma cell line, 4T1, was obtained from the American Tissue Culture Collection (ATCC, Molsheim, France). Cells were maintained in a 5% CO_2_ humidified atmosphere at 37 °C and cultured in Roswell Park Memorial Institute medium (RPMI) 1640 supplemented with 10% (*v*/*v*) fetal calf serum (Dutscher, Brumath, France). Cells were routinely tested for mycoplasma contamination using the Mycoalert Mycoplasma Detection Kit (Lonza, Levallois-Perret, France).

### 2.2. Drugs, Antibodies, and Chemical Reagents

Cisplatin (CDDP) was obtained from Sigma-Aldrich laboratory (St. Quentin Fallavier, France) and was prepared in RPMI medium; the tested antibodies included anti-poly(Adenosine DiPhosphate (ADP)-ribose)-polymerase (PARP) antibody, cleaved PARP antibody, anti-caspase-3, cleaved caspase-3, anti-caspase-7, anti-caspase-8, Bax, Bak, Bcl-2, Bcl-xl, Bid, anti-caspase-9, HSC 70, cytochrome C, Smac/Diablo, Cox IV, and β-actin, and are referenced in Supplementary Table S2, along with caspase inhibitors Q-VD-OPh (Selleckchem, Souffelweyersheim, France).

### 2.3. Preparation of Ephedra alata Extract

*Ephedra alata* was collected from the Sahara of Tataouine, a region situated in the southeast of Tunisia, in February 2017. A total of 100 g of dried powder of the aerial part of the plant were macerated into methanol (1L) for 7 days at room temperature [31]. The filtrate was concentrated by rotary evaporator under reduced pressure at 40 °C to obtain methanol extract. In order to protect our extract from oxidation, the extract was mixed with methanol in a nitrogen-closed black container to protect it from light and air contact. To facilitate the extraction procedure, the container was placed on a magnetic stirrer every day at room temperature and stored overnight at 4 °C. Moreover, this extraction process does not alter the composition of the plant. The methanol was evaporated to dryness at room temperature to produce the crude extract, which was collected and stored at −4 °C for further tests. The yield of extraction was 18.5%. Then, for cell experiments, E.A. was diluted in Dimethyl Sulfoxide (DMSO), and all control and treated cells received the same volume of DMSO (0.1%).

### 2.4. LC–MS/MS Analysis

LC-MS/MS analysis was performed on a Thermo Finnigan (Villebon sur Yvette, France) LCQ Advantage ion trap mass spectrometer with an Electrospray Ionisation (ESI) source coupled to a Thermo Scientific Accela HPLC system (MS pump plus, autosampler, and PDA detector plus) with an EC 150/2 Nucleodur 100-3 C18ec column (Macherey-Nagel, Hoerdt, France). A gradient of water and acetonitrile (ACN) was applied from 5% to 30% ACN in 60 min and from 30% to 90% ACN in another 60 min at 30 °C. The flow rate was 0.3 mL/min. The injection volume was about 25 µL. All samples were measured in the positive and negative mode. The MS was operated with a capillary voltage of 10 V, source temperature of 240 °C, and high purity nitrogen as a sheath and auxiliary gas at a flow rate of 70 and 10 (arbitrary units), respectively. The ion was detected in a mass range of 50–2000 *m*/*z*, and collision energy of 35% was used in MS/MS for fragmentation. Data acquisitions were executed by Xcalibur 2.0.7 (Thermo Scientific, Illkirch-Graffenstaden, France).

### 2.5. Cell Viability Assays

4T1 cells were seeded in sextuplicate into 96-well plates at a density of 5000 cells per well in 100 μL of medium and allowed to attach overnight. Cells were treated with increasing concentrations of E.A. and CDDP for 24, 48, and 72 h or their combinations for 48 h. At the end of treatment, cells were washed with phosphate-buffered saline (PBS), stained for 5 min with a crystal violet solution (0.5% (*w*/*v*) crystal violet in 25% (*v*/*v*) methanol), and then gently rinsed with water. Absorbances were read at 540 nm with a Biochrom Asys UVM 340 spectrophotometer following extraction of the dye by 0.1 M sodium citrate in 50% ethanol. The inhibitory concentrations of 50% (IC_50_) were calculated using a four-parameter nonlinear regression with GraphPad Prism version 6 software (GraphPad software, La Jolla California USA).

### 2.6. Combination Index Analysis

Drug interaction was determined using the method of Chou-Talalay [32,33]. Briefly, cells were plated into 96-well plates and pre-treated for 24 h with increasing concentrations of E.A. (starting concentration 100 µg/mL; 1:2 serial dilutions). Then after, increasing concentrations of cisplatin (CDDP, starting concentration 100 µg/mL; 1:2 serial dilutions) were added to wells and cells were incubated for 48 h before crystal violet staining as described for cell viability assays. The synergism, additivity or antagonism in the different combinations of the two drugs were calculated with Compusyn version 1.0 software (ComboSyn, Inc. Paramus, NJ, USA), on the basis of the multiple drug effect equation and quantification by the combination index (CI). CI was quantified with the following equation: CI = (dA/DA) + (dB/DB), where DA and DB represent the dose of drug 1 and drug 2 in the combination required to achieve the same efficacy as those of drug 1 (dA) and drug 2 (dB) when used alone [32]. The results CI > 1, CI = 1, and CI < 1 indicate antagonistic, additive, and synergistic effect, respectively. Dose-effect curve, fractional inhibition (Fa)–CI plot, and normalized isobologram were generated from CompuSyn (Combo Syn, Inc., Paramus, NJ. 07652 USA).

### 2.7. Apoptosis Analysis

4T1 cells were untreated (Co) or treated with 6 µg/mL of E.A. and/or 5 or 10 µM CDDP for 48 h. For E.A./CDDP combination, 4T1 cells were pretreated with 6 µg/mL of E.A. during 24 h, then with 5 or 10 µM CDDP for the last 48 h. Apoptosis was identified by staining the nuclear chromatin of trypsinized cells with 1 µg/mL Hoechst 33342 (Sigma Aldrich, St. Quentin Fallavier, France) for 15 min at 37 °C. The percentage of apoptotic cells was determined by analyzing 300 cells from randomly selected fields.

### 2.8. Flow Cytometry Analysis of Apoptosis

Apoptosis was determined using annexin V-FITC and 7-aminoactinomycin D (7AAD) staining from BD Biosciences (Le Pont de Claix, France) according to the manufacturer’s instructions. Cells were seeded in triplicate into 6-well plates (1.5 × 10^5^ cells per well) 24 h before treatment. The following day, 4T1 cells were untreated (Co) or treated with 6 µg/mL of E.A. and 5 or 10 µM CDDP for 48 h. For E.A./CDDP combination, 4T1 cells were pretreated with 6 µg/mL of E.A. for 24 h, then with 5 µM or 10 µM CDDP for the last 48 h. At the end of treatment, cells were harvested, washed twice with cold PBS, and then incubated for 15 min at room temperature with a mixture of annexin V-FITC/7AAD (5 µL each per sample) in 50 µL of binding buffer. At the end of the incubation, 100 µL of binding buffer was added, and cells were kept on ice until analysis. Annexin V binding and 7AAD incorporation were detected with an LSRII flow cytometer (BD Biosciences, Le Pont de Claix, France) and analyzed using FlowJo software (Tristar, Ashland, OR, USA).

### 2.9. Western Blotting

Cells were seeded into 25 cm^2^ flasks 24 h before treatment. The following day, 4T1 cells were untreated (Co) or treated with 6 µg/mL of E.A. and/or 5 or 10 µM CDDP for 48 h. For E.A./CDDP combination, 4T1 cells were pretreated with 6 µg/mL of E.A. during 24 h, then with 5 or 10 µM CDDP for the last 48 h. After treatment cells were lysed in RIPA buffer (50 mM Tris, 150 mM NaCl, 0.5% sodium deoxycholate, 1% Nonidet^TM^ P40 (NP40), 2 mM Ethylenediamine tetraacetic acid (EDTA), 50 mM Sodium Fluoride (NaF), 100 μM Phenylmethylsulfonyl fluoride (PMSF); pH 8) containing complete ultra-protease/phosphatase inhibitor (Roche, Boulogne-Billancourt, France). Protein concentration was determined using the Bradford method. About 50 µg of proteins per lane was resolved by SDS-PAGE and transferred to nitrocellulose membranes (Amersham, Les Ulis, France). Blots were then saturated in 5% milk (1 h at room temperature) before overnight incubation at 4 °C with specific primary antibodies. All primary antibodies were diluted at 1:1000 in 5% w/v non-fat milk or 5% Bovine Serum albumin (BSA). Primary antibodies were detected using horseradish peroxidase (HRP)-conjugated appropriate secondary antibodies (Cell Signaling Technologies, Ozyme, Saint-Cyr-l’Ecole, France) followed by exposure to Enhanced Chemiluminescence (ECL) (Santa Cruz Biotechnology, Heidelberg, Germany). A signal was acquired with a ChemiDoc^TM^ XRS+ imaging system (Biorad, Marnes-la-Coquette, France), and blots were analyzed with Image Lab software 5.1.2 (Biorad, Marnes-la-Coquette, France), as previously described [34].

### 2.10. Preparation of Mitochondrial and Cytosolic Protein Extracts

Cells were seeded into 25 cm^2^ flasks 24 h before treatment. The following day, 4T1 cells were untreated (Co) or treated with 6 µg/mL of E.A. and 5 and/or 10 µM CDDP for 48 h. For E.A./CDDP combination, 4T1 cells were pretreated with 6 µg/mL of E.A. for 24 h, then with 5 µM or 10 µM CDDP for the last 48 h. After treatment, subcellular fractions of 4T1 cells were extracted by the methods described in the manufacturer’s instructions for the Mitochondrial/Cytosol Fractionation Kit (Enzo Life Sciences, Villeurbanne, France). Briefly, treated cells were collected by centrifuge at 4 °C. The cells were suspended with cytosol extraction buffer mixture containing Dithiothreitol (DTT) and protease inhibitors, and incubated on ice for 10 min. Then, the cells were homogenized in an ice tissue grinder and centrifuged again at 4 °C. The supernatant, corresponding to the cytosolic fraction, was collected and stored at −80 °C. The pellet was suspended in mitochondrial extraction buffer, vortexed for 10 min, and saved as the mitochondrial fraction at −80 °C.

### 2.11. Mouse Strain and Tumor Growth Experiments

A total of 24 female BALB/c mice (8 weeks old and weighing ≈25 g) were housed according to the Council of the European Communities (86/609/EEC; November 24th 1986) Directives regulating the welfare of experimental animals, and experiments were approved by the Life Sciences and Health Research Ethics Committee (cer-svs) of the Institute of Biotechnology (University of Monastir, Tunisia; ethical approval no. 2019/02/I/CER-SVS/ISBM; 9 January, 2019). The mice were maintained in a pathogen-free environment (24 °C and 50% humidity) on a 12 h light/12 h dark cycle, with food and water supplied ad libitum throughout the experimental period. Mice were allowed to acclimatize under the laboratory condition for 1 week before being subjected. Tumor formation was initiated by injecting 1 × 10^6^ 4T1 cells into the mammary fat pad region of mice, with this corresponding to day 1. Treatment started from the seventh day, and the mice were then randomly divided in four groups: vehicle (as a control), E.A., CDDP, and combination E.A. of and CDDP treatment (*n* = 6 per group). For all groups, DMSO was used at the final concentration 0.1% and the control group received a saline solution with 0.1% of DMSO (sterile DMSO (Sigma Aldrich, St. Quentin Fallavier, France, reference 67-68-5)). Group 1 mice were treated with E.A. diluted in DMSO, which was administered three times per week by intraperitoneal injection (150 mg/kg). Group 2 mice were treated with cisplatin in the same vehicle of control, which was administered once a week by intraperitoneal injection (20 mg/kg). Group 3 mice received a combination treatment with the same concentrations of CDDP and E.A. as in groups 1 and 2. The remaining six mice in the control group were administered sterile saline with the same timing and dosing schedule as that used for the other treatment groups. The tumor volumes and body weights of mice were measured throughout the study. Tumor volume was calculated using the following formula: TV (mm^3^) = d^2^ × D/2, where d and D are the shortest and the longest diameters, respectively.

### 2.12. Biochemical Assays and Analysis

Trunk blood samples were collected from the sacrificed mice. The serum samples were analyzed by the Biochemistry Department laboratory of the University Hospital Center (UHC) Farhat Hached (Sousse, Tunisia). Aspartate transaminase (AST), alanine transaminase (ALT), creatinine (CR), and phosphatase alkaline (PAL) were determined by automated analysis using a commercial Cobas Integra kit (Roche, Boulogne-Billancourt, France).

### 2.13. Statistical Analysis

The data are expressed as mean ± SD. Statistical comparisons among groups were analyzed using one-way and two-way analysis of variance (ANOVA), followed by Tukey’s multiple comparison test. Statistical significance was considered for *p*-value < 0.05.

## 3. Results

### 3.1. Identification and Quantification of Phenolic Compounds in E.A. Extracts

Many authors have been able to highlight the great diversity of compounds that may be present in *Ephedra* species, in particular, a wide variety of polyphenolic compounds, but also, depending on the species, the presence of ephedrine or pseudoephedrine, which could limit their use due to the effects potentially harmful of these last two compounds. Recently, Ibragic et al. (2015) were able to show that among the species of *Ephedra*, *Ephedra alata* (E.A.) possessed a significant content of polyphenols and, more particularly, flavonoids, compared to its low content of alkaloids (ephedrine and pseudoephedrine) [20]. However, this content seems very dependent upon various factors such as the amount of rainfall, soil characteristics, harvesting, and storage conditions of the plant, as well as the analytical quantification method. In our study, in order to assess the potential effects of an E.A. extract on breast cancer cells, we first determined the quantitative and qualitative composition of E.A., which was collected from the Sahara of Tataouine, a region situated in the southeast of Tunisia, in February 2017. As indicated in the Materials and Methods section, the methanol extract obtained from the aerial part of E.A. was analyzed by LC-MS-MS. The results obtained revealed 20 secondary metabolites representing different classes of compounds, including flavonoids, phenolic acids, and proanthocyanidins with different levels (Table 1). All the compounds were identified on the basis of molecular ions and subsequent fragment ions (Figure S1 and Table S1). More specifically, we identified some phenolic acids such as caffeic acid, and derivatives, citric acid, ferulic acid, syringic acid, and its derivative syringic acid hexoside, as well as a high content of quercetin and derivatives (i.e., quercetin dihexoside, quercetin-3-*O*-galactoside, 6-hydroxyquercetin-3-*O*-di-hexose, quercetin 3-*O*-[6”-(3-hydroxyl-3-methylglutaryl)-β-d-galactoside], which present the most important level, quercetin 3-*O*-rhamnoside 7-*O*-rhamnoside). Besides quercetin, there are other derivatives of myricetin, these being myricetin hexoside and myricetin-3-*O*-glucoside, as well as other compounds such as luteolin-8-*C*-β-d-glucopyranoside, vicenin-2, isoorientin-4-*O*-glucoside, and Kaempferol-*O*-di-deoxyhexoside. One proanthocyanidin was identified as gallocatechin (Table 1).

### 3.2. E.A. Extract Decreased Breast Cancer Cells Viability

**Figure 1:**
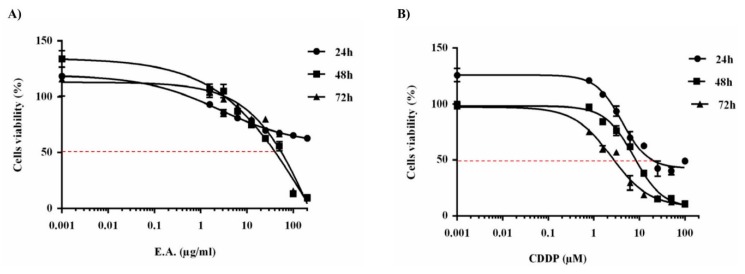
Concentration-dependent inhibition of breast cancer cell viability by E.A. extract. After 24 h of culture, breast cancer cells 4T1 were exposed with increasing concentrations of (**A**) E.A. (from 0 to 100 μg/mL) or (**B**) cisplatin (CDDP) (0 to 100 µM) at 37 °C for 24 h (●), 48 h (■), and 72 h (▲). The percentage of cell viability was determined by crystal violet assay. Results are expressed as percentage of control (mean ± standard deviation of three independent experiments with *n* = 6).

To determine whether E.A. extract exerts a potential anticancer action, we first exposed 4T1 breast cancer cells to increasing amounts of E.A. (0 to 100 µg/mL) diluted in DMSO at 0.1% for 24, 48, and 72 h prior to cell viability assays (Figure 1). As revealed by the cytotoxic curves, E.A. displayed a decrease of cell viability in 4T1 cancer cells for 48 and 72 h in a dose-dependent manner (Figure 1A). The concentrations inhibiting 50% of cell viability (IC_50_) showed an IC_50_ for E.A. of 65 and 30 µg/mL for time incubations of 48 and 72 h, respectively (Table 2). In a similar manner, a classical drug, such as cisplatin (CDDP), can induce cytotoxic effects after 24 h of treatment, increasing in a time-dependent manner (Figure 1B). This antiproliferative activity of E.A. is similar to that in which we observe with other natural compounds whose cytotoxic activity is often less than that of conventional anticancer agents [35,36,37]. However, the interest of these molecules often resides in the fact that the latter have the capacity to sensitize tumor cells to therapeutic agents, as we have been able to dismantle it previously with an emblematic polyphenol, resveratrol [13,14].

### 3.3. E.A. Synergized with CDDP to Reduce 4T1 Breast Cancer Cell Viability

**Figure 2:**
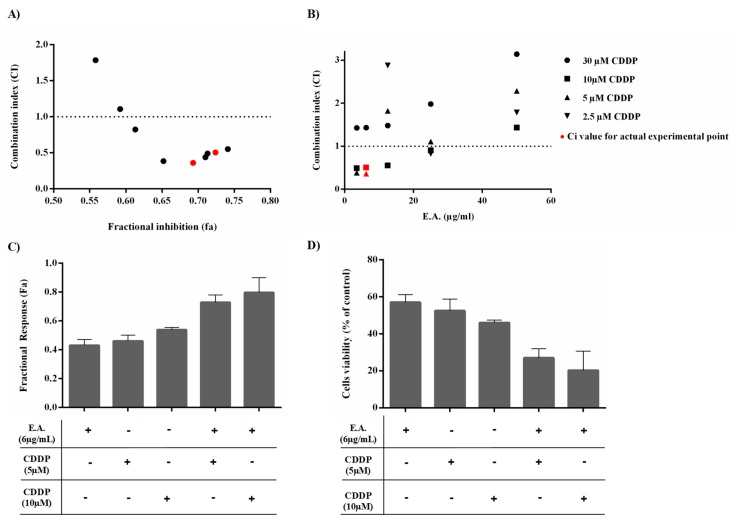
Determination of the combinatory effect of E.A. with CDDP on 4T1 cancer cell viability. 4T1 tumoral cells were pre-treated for 24 h with increasing concentrations of E.A. and then treated for 48 h with increasing concentrations of CDDP. The relative amount of adherent (alive) cells was thereafter quantified by crystal violet assay. (**A**,**B**) The combination index (CI) plots were constructed from the data obtained from the CompuSyn report for E.A. extract and CDDP combinations. CI values indicate additive (CI = 1), synergistic (CI < 1), and antagonistic (CI > 1) effects. The horizontal dash lines at CI = 1 were drawn, and the actual experimental points are indicated in red. (**C**) Fractional response (Fa) and cell viability (**D**) for synergistic combinations. Results shown as means ± SD for triplicate assay samples reproduced independently at least three times.

In order to evaluate the potential use of E.A. as a chemosensitizing agent that is able to counteract chemoresistance, we searched for a potential synergistic effect of E.A. extract with a classical anticancer agent widely used in the treatment of breast cancer, such as cisplatin (CDDP) [38]. Breast cancer cells 4T1 were thus pre-incubated 24 h with increasing concentrations of E.A., and were then treated with increasing concentrations of CDDP for 48 h before determining cell viability by crystal violet staining. Interactions between E.A. and CDDP were assessed by calculating the combination index (CI) by the Chou–Talalay method, where CI > 1 indicates antagonism, CI = 1 additivity, and CI < 1 synergism. Mean CI from data points was calculated and plotted against fractional inhibition (Fa), representing growth inhibition potency of drug combination. The Fa < 0.5 were excluded, as they indicated less than 50% growth inhibition, which is less relevant for anticancer agents to therapy. Synergism between E.A. and CDDP was recognized (CI < 1; Figure 2A), although not for all drug combinations, which resulted mostly in antagonism (CI values > 1). Indeed, synergistic effects were observed only at 6 µg/mL E.A. combined with either 5 or 10 µM CDDP (Figure 2B). These drug combinations were associated with greater Fa values and greater reduction in cancer cell viability than conventional agents alone (Figure 2C,D). Thus, due to this very interesting result, in the remainder of the present study, E.A. extract was used in at a nontoxic concentration and subsequently as a potential chemosensitizer to enhance the activity of cisplatin in the tumor cells, while limiting any undesired toxicity or side effects.

### 3.4. E.A. Synergized with CDDP to Enhance Apoptosis in 4T1 Breast Cancer Cells

**Figure 3:**
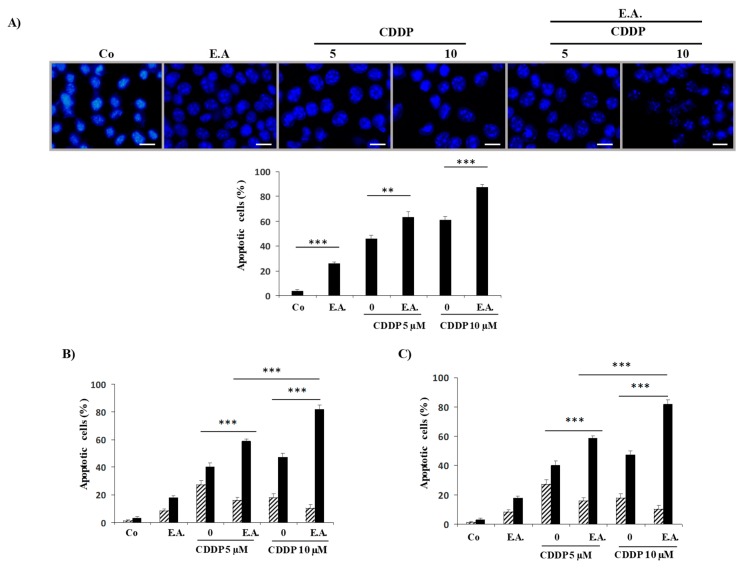
E.A. sensitized breast cancer cells to CDDP-induced apoptosis. (**A**) 4T1 cells were untreated (Co) or treated with 6 µg/mL of E.A. (E.A.) and 5 or 10 µM CDDP for 48 h. For the E.A./CDDP combination, 4T1 cells were pretreated with 6 µg/mL of E.A. for 24 h, then with 5 µM or 10 µM CDDP for the last 48 h. The percentage of cells with a condensed nuclear chromatin, as identified by Hoechst 33342 staining (scale bar = 10 µm), was measured at the end of cell treatment (mean ± SD of three independent experiments; 300 cells per point). (**B**) 4T1 cells were treated as described in (A) and then stained with annexin V/aminoactinomycin D (7AAD) at the end of treatments. Percentage of early apoptotic cells (identified as the annexin V-positive/7AAD negative population, in hatched grey) and the late apoptotic cells (identified as the annexin V-positive/7AAD positive population, black bar) were calculated. The data are means ± standard deviation of three independent experiments; *p*-values were determined by the multiple ANOVA test. * *p* < 0.05, ** *p* < 0.01, *** *p* < 0.001. (**C**) 4T1 cells were pretreated for 2 h with a pan-caspase inhibitor, Q-VD (10 µM), before treatment, as in (**A**). Then, cells were harvested and stained as in (**B**) with annexin V/7AAD at the end of treatments. The percentage of early and late apoptosis were determined as in (**B**).

The cytotoxic effect of E.A. on 4T1 cancer cells is associated with an induction of a cell death by apoptosis. Indeed, cell staining with Hoechst 33342 demonstrated that E.A. induced an increase in the nucleus size that preceded the appearance of characteristic apoptosis changes, that is, the condensation and fragmentation of the nuclear chromatin (Figure 3A). The quantification of cells with a condensed nuclear chromatin revealed that both E.A. and CDDP induced apoptosis, but that an E.A./CDDP combination induced a strong induction of breast cancer cell apoptosis. This induction by E.A. corroborates the cytotoxic effect previously observed, and similarly with CDDP, we observed a higher induction of apoptosis (Figure 3A). However, the main information observed was the strong induction of apoptosis with the combination E.A./CDDP at the concentrations that we have determined previously as acting synergistically on cell viability (Figure 3A). To better analyze E.A./CDDP combination impact on apoptotic process, breast cancer cells were double-stained with annexin V/7AAD after pretreatment with the E.A. (6 µg/mL) for 24 h before CDDP (5 and 10 µM) for 48 h. It appeared to be the case that E.A. pretreatment cooperated positively with the anticancer drug CDDP to induce particularly late apoptosis, which was significantly more effective than treatment with the drug alone (Figure 3B). These inductions of late apoptosis were dramatically inhibited by co-treatment with an irreversible broad-spectrum caspase inhibitor, Q-VD (10 µM), suggesting caspase involvement in the death process induced by both E.A. or by the combination E.A./CDDP (Figure 3C).

### 3.5. E.A. Sensitized Breast Cancer Cells to CDDP-Mediated Caspase Activation

**Figure 4:**
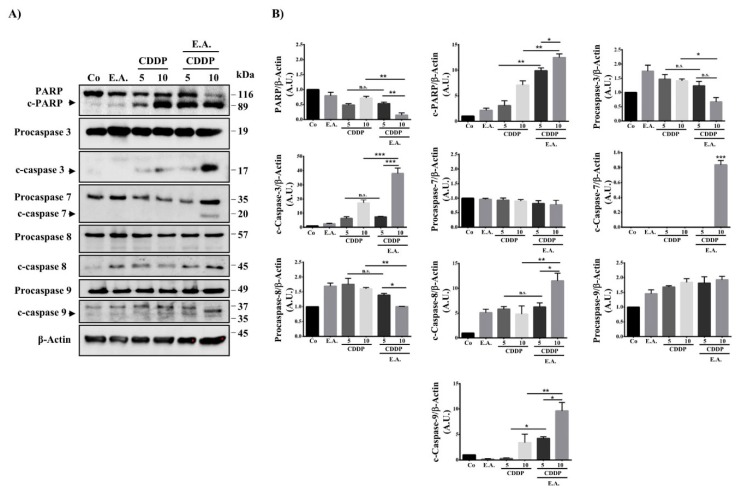
E.A./CDDP combination induced caspase activation in breast cancer cells. (**A**) Immunoblotting analysis of indicated proteins in 4T1 cells exposed for 48 h to the vehicle (Co) or to E.A. (6 µg/mL), CDPP (5 and 10 µM), or to the combination of E.A (6 µg/mL) and CDDP (5 or 10 µM). An anti-actin antibody (Ab) was used for loading control. One representative of three independent experiments. (**B**) Densitometry quantification of Western blotting obtained in (**A**). PARP: poly(ADP-ribose)-polymerase and c-PARP: cleaved- poly(ADP-ribose)-polymerase. The data are means ± standard deviation of three independent experiments; *p*-values were determined by the multiple ANOVA test. * *p* < 0.05, ** *p* < 0.01, *** *p* < 0.001.

The main actors of apoptosis are represented by a family of cysteine aspartyl proteases, namely, caspases, which are normally present as procaspases and are inactive zymogens. In order for a cell to undergo apoptosis, procaspases must become activated via cleavage or dimerization [39]. Immunoblots demonstrated that *M_r_* 32,0000 proform of caspase-3, the *M_r_* 36,000 proform of caspase-7, the *M_r_* 55,000 proform of caspase-8, and the *M_r_* 47,000 proform of caspase-9 were cleaved into their active fragments significantly during the death process induced by the different treatments (Figure 4A,B). The quantification of immunoblotting highlights that a pretreatment of 24 h with 6 µg/mL of EA and a treatment of 48 h with 10 µM of CDDP (E.A. 6 µg/mL/ CDDP 10 µM combination) was the most effective in leading caspase activation (Figure 4B). The role of these proteases in E.A./CDDP combination-induced apoptosis was further suggested by the observation that the nuclear DNA repair enzyme poly(ADP-ribose)-polymerase (PARP) was cleaved into an *N*-terminal 89 kDa fragment in treated breast cancer cells (Figure 4A,B).

### 3.6. E.A./CDDP Combination-Induced Apoptosis through a Modulation of Bcl-2 Proteins Family

**Figure 5:**
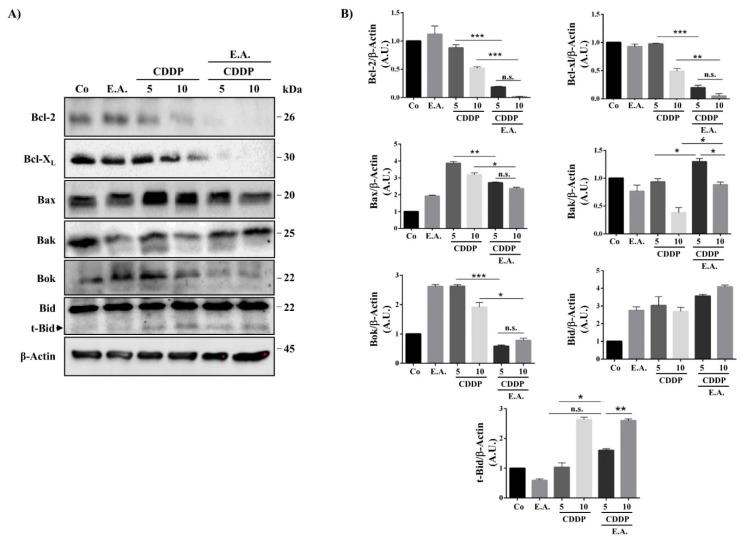
E.A./CDDP combination synergized to strongly affect Bcl-2 family protein expression in breast cancer cells. (**A**) Immunoblotting analysis of indicated proteins in 4T1 cells exposed for 48 h to the vehicle (Co) or to E.A. (6 µg/mL), CDPP (5 and 10 µM), or to the combination of E.A (6 µg/mL) and CDDP (5 or 10 µM). An anti-actin Ab was used for loading control. One representative of three independent experiments. (**B**) Densitometry quantification of Western blotting obtained in (**A**). The data are means ± standard deviation of three independent experiments; *p*-values were determined by the multiple ANOVA test. * *p* < 0.05, ** *p* < 0.01, *** *p* < 0.001. Bcl-2, B-cell lymphoma 2 protein; Bcl-XL, B-cell lymphoma-extra large protein; Bax, Bcl-2-associated X protein; Bak, Bcl-2 homologous antagonist/killer protein; Bok, Bcl-2-related ovarian killer protein; Bid, BH3 interacting-domain death agonist; t-Bid, truncated BH3 interacting-domain death agonist.

Usually, two main death pathways are described: the “extrinsic pathway” involving death receptors and activation of caspase-8, and the “intrinsic pathway” that involves the mitochondria. This last pathway is mainly activated by anticancer agents through a modulation of anti-apoptotic proteins such as Bcl-2 itself, as well as Bcl-X_L_, which prevent the release of pro-apoptotic molecules such as cytochrome C or Smac/Diablo. To further explore the mitochondria involvement in E.A./CDDP combination-induced apoptosis in 4T1 cells, we analyzed the effect of compounds alone and with the E.A./CDDP combination on the expression of Bcl-2 and related proteins that control the permeabilisation of the mitochondrial outer membrane. Immunoblotting reveals that exposure of 4T1 cells to both combinations of E.A./CDDP induced a dramatically decrease in the expression of anti-apoptotic Bcl-2 and Bcl-X_L_ proteins without a significant modulation of pro-apoptotic proteins Bax and Bak (Figure 5A,B). Very interestingly, E.A./CDDP combination completely blunted Bok expression in the cancer cells treated, suggesting that this Bcl-2 ovarian killer, which controlled a non-canonical apoptosis pathway, is not involved in the death process-induced by the combination. At the inverse, as shown by the quantitative analysis of immunoblots, we observed a statistical increase of the truncated and active form of Bid, t-Bid, which ensured the crosstalk between extrinsic and intrinsic pathways (Figure 5B).

### 3.7. E.A./CDDP Combination Induced Smac/Diablo and Cytochrome C Release from the Mitochondria

**Figure 6:**
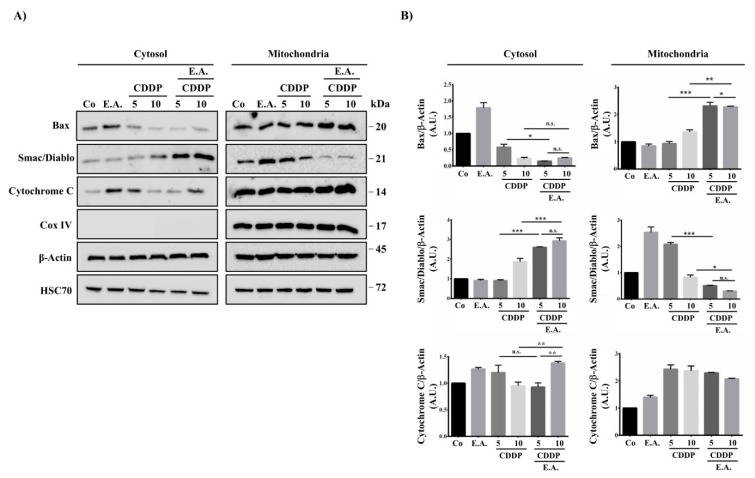
E.A./CDDP combination synergized to activate intrinsic death pathway in breast cancer cells. (**A**) Immunoblotting analysis of indicated proteins in 4T1 cells exposed for 48 h to the vehicle (Co) or to E.A. (6 µg/mL), CDPP (5 and 10 µM), or to the combination of E.A (6 µg/mL) and CDDP (5 or 10 µM) in the mitochondria and the cytosol. An anti-actin antibody (Ab) was used for loading control. One representative of three independent experiments. (**B**) Densitometry quantification of Western blotting obtained in (**A**). The data are means ± standard deviation of three independent experiments; *p*-values were determined by the multiple ANOVA test. * *p* < 0.05, ** *p* < 0.01, *** *p* < 0.001. Cox IV, Cytochrome c oxidase IV; HSC-70, Heat shock 70-KDa protein cognate.

Usually, anti-apoptotic proteins block the release of pro-apoptotic soluble molecules from the intermembrane space of these organelles to the cytosol, whereas pro-apoptotic proteins such as Bak and Bax migrate to the mitochondria to promote the release of cytochrome c and Smac/Diablo into the cytosol to activate the proteolytic cascade of caspases activation. We observed that E.A./CDDP combinations induced a decrease of the pro-apoptotic Bax protein in the cytosol and its accumulation in the mitochondria (Figure 6A). This observation was associated with a strong release of pro-apoptotic soluble molecule Smac/Diablo from the mitochondria to the cytosol, as compared to the control and, to a lesser extent, the cytochrome C (Figure 6A,B). These results were corroborated by the achievement of ratios of cytosol/mitochondria fractions. Indeed, as shown in the ratios in Table 3, an E.A./CDDP 10 µM combination resulted in a decrease in the ratio for the pro-apoptotic Bax protein, indicating a greater relocalization of the pro-apoptotic protein to mitochondria. At the inverse, the ratio for the Smac/Diablo protein increased very significantly with the combination of the two drugs (E.A./CDDP) compared to their use alone, thus reflecting the release of the soluble proapoptotic molecule from the intermembrane space of mitochondria to the cytosol (Table 3). Subsequently, these results are in accordance with the activation of initiator caspase-9 and the effector caspases-3 and -7 (Figure 4).

### 3.8. E.A./CDDP Combination Synergized to Prevent Murine Mammary Carcinoma Growth

**Figure 7:**
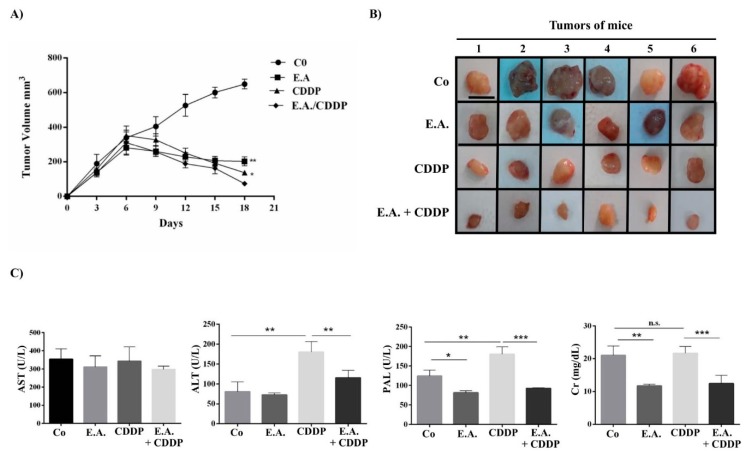
E.A./CDDP combination synergized to inhibit tumor growth and to prevent hepato- and nephrotoxicities of CDDP. (**A**) Eight-week-old BALB/c female mice were injected in the mammary fat pad region 1 × 10^6^ 4T1 cells, and then each group of mice (six per group) was treated at the seventh day with sterile saline (control group; Co), E.A. (150 mg/kg), CDDP (20 mg/kg), or with E.A. (150 mg/kg) and CDDP (20 mg/kg) combination. Tumor size in cubic millimeters over time is represented as median ± standard error of the mean (SEM). (**B**) Xenograft tumors from mice groups (six per group were harvested at day 18; scale bar = 1 cm). (**C**) Biochemical analyses of the serum from mice groups for aspartate transaminase (AST), alanine transaminase (ALT), creatinine (Cr), and phosphatase alkaline (PAL). The data are means ± standard deviation (SD) of three independent experiments; *p*-values were determined by the multiple ANOVA test. * *p* < 0.05, ** *p* < 0.01, *** *p* < 0.001.

In a last step, we sought to correlate the synergic effect of E.A./CDDP combination in vitro with the ability to prevent tumor progression. In order to demonstrate this, female BALB/c mice received subcutaneous murine tumoral breast 4T1 cells and then received a fixed amount of E.A. (150 mg/kg) three times per week, or CDDP (20 mg/kg) one time per week, or a combination of E.A. (150 mg/kg) and CDDP (20 mg/kg) (three times per week for E.A. and one time per week for CDDP), or with sterile saline solution for the control group. Our results showed for the first time that E.A. was able to delay significantly the growth of tumors as early 6 days after tumor implantation, as compared to the tumor growth of mice controls (Figure 7A,B). This difference was sharply accentuated 15 days after the injection of 4T1 cells. Importantly, the synergistic effect observed in our previous in vitro experiments was found again in our tumoral model, where E.A./CDDP combination showed a significant decrease of tumor growth as compared to E.A and CDDP treatment alone (Figure 7A,B). The side effects of CDDP that are usually associated with its antitumoral effect were found again in this in vivo experiment, where blood sample analysis revealed a high level of phosphatase alkaline (PAL), alanine transaminase (ALT), and creatinine (CR) in mice treated only with CDDP (Figure 7C). However, very surprisingly, the combination of CDDP with E.A. decreased the side effects of CDDP, as shown by a significant decrease of these three markers of CDDP toxicity (Figure 7C).

## 4. Discussion

Despite major advances in the last 10 years, whether in terms of prevention or treatment, the 5 year survival rate remains relatively low for a large number of cancers. These therapeutic failures can be the consequence of several factors, whether endogenous or exogenous. First of all, there is the acquisition of resistance mechanisms developed by tumor cells or of acquired resistance, such as the overexpression of efflux pumps or the development of mechanisms for bypassing the pathways leading to apoptosis; secondly, the tumor microenvironment also plays an important role through increased neoangiogenesis and a phenomenon of hypoxia; and finally, these therapeutic failures can be linked to the host, which can further eliminate the anticancer agent or the suffering of side effects being so great that stopping treatment is necessary. Then, faced with these therapeutic failures, for several years’, new strategies have been developed that aim to increase the effect of anti-cancer compounds and/or to reduce their secondary effects on normal cells, thus enabling the host to better withstand chemotherapy. In the present report, we studied the effect of *Ephedra alata* Decne extract (E.A.) rich in polyphenolic compounds towards breast cancer model in vitro and in vivo. We found that E.A. inhibited cell viability of 4T1 breast cancer cells and induced apoptosis in a caspase-dependent manner. This programmed cell death involved a release of pro-apoptotic soluble molecules (i.e., Smac/Diablo, cytochrome C) from the intermembrane space of mitochondria to cytosol. This antiproliferative effect contributed to significant delay of tumor growth in mice. Herein, for the first time, we highlighted the ability of E.A. to synergize with an anticancer drug such as cisplatin (CDDP). Indeed, a combination of the E.A. extract with CDDP enhanced the cell death of 4T1 breast cancer cells through a strong caspase activation, a decrease of anti-apoptotic Bcl-2 proteins, and a relocalization of pro-apoptotic molecules into the cytosol to active effector caspases such as caspases-3 and -7 to finally cleave PARP protein. All these processes favored an important antitumoral activity of E.A./CDDP combination, which inhibited tumor breast growth in mice. Very surprisingly, these in vivo experiments revealed that E.A./CDDP combination, in addition to having a synergistic effect against tumor growth, decreased some markers such as ALT, PAL, and CR. Very few studies have reported the anticancer effect of *Ephedra* in particular against breast cancer, probably because of the toxic effects due to the presence of ephedrine. Indeed, ephedrine and derivatives (i.e., pseudoephedrine, norephedrine, methylephedrine) are derived from phenyl-alanine and act on the sympathetic nervous system as a sympathomimetic [40]. In this way, food supplements containing E-type alkaloids represent a potential health risk, bearing in mind the conditions of use and often involve the removal of all over-the-counter drugs containing ephedrine. However, besides this class of alkaloids, which represent only 0.02% to 3.4% in the aerial part of *Ephedra alata* Decne, there are various polyphenolic compounds such as flavones, flavanols, bisflavanols, and carboxylic acids. These compounds provide various beneficial properties, in particular, antioxidant, anti-inflammatory, and antiproliferative activities. Quantitative and qualitative analyses have been able to show the importance of the ratios between the ephedrine content and the polyphenol content according to the *Ephedra* species considered. Subsequently, *Ephedra* species, which are avoidant of E-alkaloids and present a high level of polyphenols, could represent a potential interest due to the health benefits of polyphenol compounds. 

On the basis of these studies, we qualitatively determined the composition of a particular extract of *Ephedra alata*, which was collected from the Sahara of Tataouine, a region situated in the southeast of Tunisia, by using conventionally used methods of analysis. These have thus been able to reveal that our extract had high levels of polyphenols, including flavonoids such as quercetin in particular. This point is very important to consider for the determination of the biological effects of the extract because these compounds can exert a synergistic or an additive effect between them, as well as antagonistic effects. Indeed, during previous studies carried out on polyphenolic extracts of red wine, we were able to demonstrate that, depending on the qualitative and quantitative contents of polyphenols, we could observe a synergistic, additive or, for some, antagonistic effect [41,42]. In the present study, our extract presented a rich content of polyphenolic compounds without ephedrine or derivatives (Table 1). More interestingly, we detected a high level of quercetin and various glucoside derivatives; this particularity is important because quercetin has been extensively studied on various cancer models such as breast cancer, where this flavonoid presents remarkable antiproliferative activity [43,44]. Although some studies on different species of *Ephedra* may have shown an effect on breast cancer cells, such as *Ephedra chilensis* K Presl [45] and *Ephedra aphylla* [46], only one experimental study has reported a potential interest of *Ephedra alata* in breast cancer, where a hydro-alcoholic extract of E.A. was able to present an antiproliferative and pro-apoptotic action against the MCF-7 human breast cancer cell line [29]. In our study, we highlighted that E.A. was able to induce apoptosis through a caspase-dependent pathway involving both extrinsic and intrinsic pathways. Moreover, very interestingly, we showed for the first time the potential synergism between E.A. and CDDP, a classical anticancer drug usually used against breast cancer. This combination led to a knockdown of anti-apoptotic Bcl62 and Bcl-x_L_ protein expression with a release of cyt C and Smac/Diablo proteins from the mitochondria to the cytosol. This point is important, as the anti-apoptotic proteins of the Bcl-2 family contribute to the survival advantage. Thus, by neutralizing anti-apoptotic proteins, the E.A./CDDP combination modified the Bax/Bcl-2 and Bax/Bcl-x_L_ ratio and promoted apoptosis in breast cancer cells. Subsequently, procaspase-9 was activated and caspases-3 and -7, which are critical mediators of mitochondrial events of apoptosis [39], can exert their proteolytic action on PARP to lead the final step of apoptosis. Very interestingly, we detected under treatment with the E.A./CDDP combination the truncated form of Bid (t-Bid), which is a member of the Bcl-2 Homology (BH) 3 domain-only subgroup of Bcl-2 family. This t-Bid protein could function as a membrane-targeted death ligand in which an intact BH3 domain is required for cytochrome c release [47]. In the extrinsic pathway, activation of caspase-8, an initiator of the downstream apoptotic process that includes the activation of caspase-3, -6, and -7, was found to be able to cleave Bid. Once cleaved, t-Bid targets mitochondria to promotes apoptosis [48]. Thus, the E.A./CDDP combination was able to activate caspase-8 and promote cleavage of Bid to enhance apoptosis.

All together, these properties contributed strongly to the inhibition of tumor breast growth in mice with the E.A./combination and, very surprisingly, this combination decreased the toxic effects induced by CDDP. Indeed, the use of CDDPP is usually associated with side effects such as nephrotoxicity or hepatotoxicity. Indeed, the onset of renal insufficiency begins several days after the dose of cisplatin, as revealed by increases in the serum creatinine [49]. In the same way, CDDP has been associated with a low rate of serum enzyme elevations during therapy (i.e., PAL, ALT, AST), reflecting a hepatotoxicity [50]. The addition of E.A. decreases the serum creatinine level significantly in phosphatase alkaline (PAL), as well as alanine amino transferase (ALT).

## 5. Conclusions

**Figure 8:**
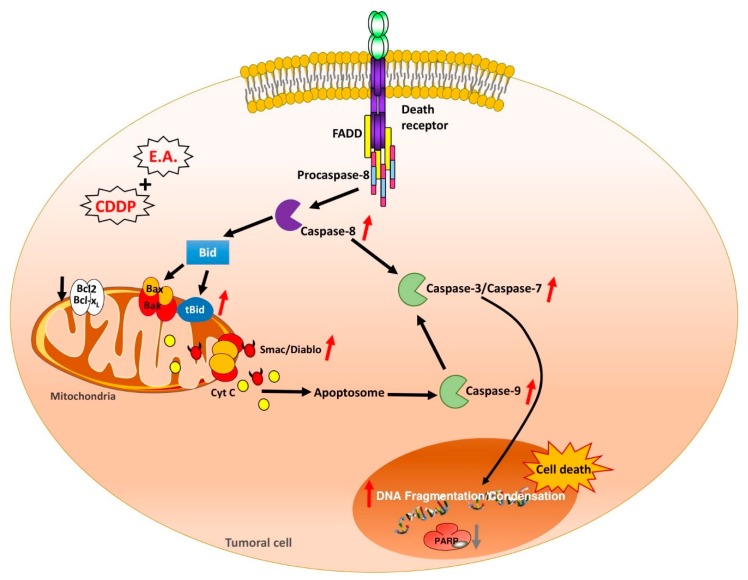
Key regulators modulated by an E.A./CDDP combination in breast cancer cells. E.A./CDDP acts on the intrinsic pathway of apoptosis by decreasing the expression of BCL-2 and BCL-x_L_ anti-apoptotic proteins and by increasing the expression of Bax and Bak proteins. The relocalization of Bax into mitochondria would allow the release in the cytosol of pro-apoptotic molecules contained in the mitochondrial intermembrane space, allowing then the activation of the procaspase-9 in caspase-9, which, once activated, will turn on caspase-3. Also this E.A./CDDP combination is able to activate capsase-8, one of the apoptotic markers of the extrinsic pathway allowing both the cleavage of the Bid protein, where Bid truncate will proceed to activate the mitochondrial pathway and the cleavage of procaspase-3 into active caspase-3, which will target the cleavage of PARP and then lead to cell death (FADD: Fas-Associated protein with Death Domain).

These first results highlight the interest of E.A. extract in order to enhance the anticancer activity of cisplatin in breast cancer treatment and its potential use as a chemosensitizer in association with CDDP to both decrease its nephro- and hepatotoxic effects and to enhance its anticancer activity (Figure 8). Thus, additional studies must now be conducted on a wider panel of breast cancers of different stages and different grades in order to better understand the chemosensitization by E.A., but also to determine if E.A. can act in synergy with other anticancer agents, by which it could enhance the activity by modulating one or more mechanisms of resistance.

## Figures and Tables

**Table 1:** Qualitative and quantitative content of *Ephedra alata* Decne extract (E.A.).

Compound	Content (%)
Caffeic acid derivative	0.54
Citric acid	0.28
Syringic acid hexoside	0.70
Syringic acid	0.73
Gallocatechin	0.40
Ferulic acid	0.19
Quercetin dihexoside	1.02
Vicenin-2	9.33
Quercetin-3-*O*-galactoside	1.95
Isoorientin-4-*O*-glucoside	1.74
6-Hydroxyquercetin-3-*O*-di-hexose	14.67
Quercetin 3-*O*-glucoside	2.48
Luteolin-8-*C*-β-d-glucopyranoside	0.18
Quercetin	19.85
Myricetin hexoside	8.59
Quercetin 3-*O*-[6”-(3-hydroxyl-3-methylglutaryl)-β-d-galactoside]	24.48
Myricetin-3-*O*-glucoside	3.05
Quercetin 3-*O*-rhamnoside	0.76
7-*O*-rhamnoside	
Kaempferol-*O*-di-deoxyhexoside	1.08

**Table 2:** IC_50_ values determined from cell viability assays for E.A. and CDDP on the 4T1 breast cancer cells at the different times of treatment.

IC_50_	24 h	48 h	72 h
E.A.	-	65 µg/mL	30 µg/mL
CDDP	20 µM	10 µM	3 µM

**Table 3:** Ratio of cytosol/mitochondria fractions after treatment on the 4T1 breast cancer cells.

	Ratio Cytosol/Mitochondria Fractions		
	Bax	Smac/Diablo	Cytochrome c
E.A.	2.1	0.38	0.91
CCDP 5 µM	0.62	0.43	0.45
CCDP 10 µM	0.25	0.90	0.55
E.A. + CCDP 5 µM	0.12	5.10	0.41
E.A. + CCDP 10 µM	0.10	9.69	0.66

## Supplementary Materials

The following are available online at https://www.mdpi.com/2073-4409/9/2/362/s1, Figure S1. LC-MS-MS Analysis of the main compounds identified; Figure S2. ESI-MS/MS profiles of identified compounds; Table S1. Summary of the properties of the compounds detected in an *Ephedra alata* extract of by LC-MS/MS; Table S2. Antibodies used in the study.
