# Supplementary material for: A New Highlight of Ephedra alata Decne Properties as Potential Adjuvant in Combination with Cisplatin to Induce Cell Death of 4T1 Breast Cancer Cells In Vitro and In Vivo
Source: Cells. 2020 Feb 4;9(2):362. doi: 10.3390/cells9020362 (PMC7072491; doi:10.3390/cells9020362)

Supplementary Figure 1

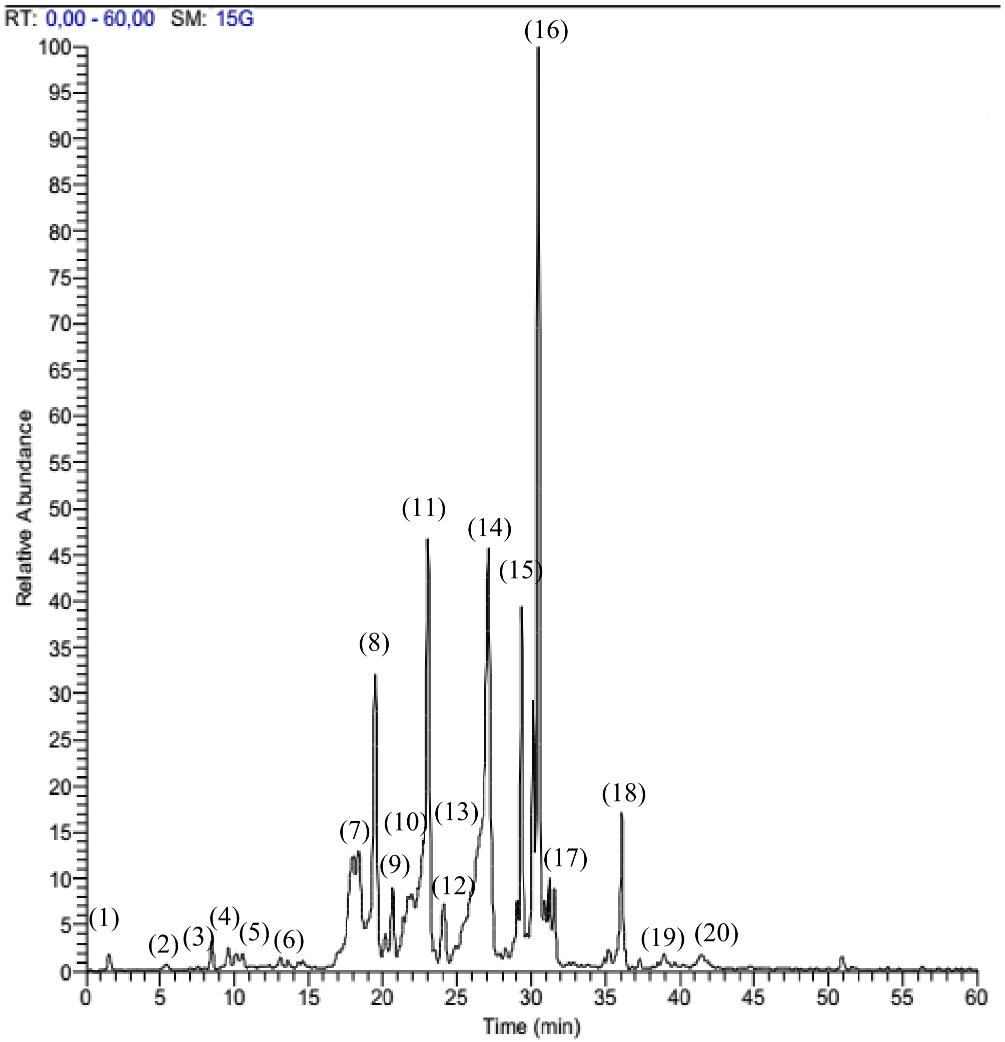

Supplementary Table I. Summary of the properties of the compounds detected in an *Ephedra alata* extract of by LC-MS/MS.

Peak	RT (min)	[M+H]- (m/Z)	HPLC ms2	Identified compounds
**1**	1.51	377	341, 215, 179, 332	Caffeic acid derivative
**2**	5.22	191	173, 111, 129	Citric acid
**3**	8.45	359	197, 182,167	Syringic acid hexoside
**4**	9.54	197	182, 167,153	Syringic acid
**5**	10.52	305	179, 219, 261, 105, 137,125	Gallocatechin
**6**	13.65	193	149, 178,134	Ferulic acid
**7**	18.39	625	463 ,301	Quercetin dihexoside
**8**	19.38	593	473, 503. , 353, 575 , 383	Vicenin-2
**9**	22.18	463	300	Quercetin-3-O-galactoside
**10**	22.43	609	489, 447	Isoorientin-4 -O-glucoside
**11**	23.21	641	479, 317	6-Hydroxyquercetin-3-O-di-hexose
**12**	24.86	463	301,343	Quercetin 3-O-glucoside
**13**	25.5	447	285	Luteolin-8-C-β-D-glucopyranoside (Orientin)
**14**	26.65	301	301	Quercetin
**15**	29.43	479	317	Myricetin hexoside
**16**	30.45	607	463 , 301, 505, 545	Quercetin 3-O-[6"-(3-hydroxyl-3-methylglutaryl)-β-D-galactoside]
**17**	30 .93	479	317,313 ,151 ,179	Myricetin-3-O-glucoside
**18**	36.13	756	593, 594	Unknown
**19**	38 .9	593	301,300, 447	Quercetin 3-*O*-rhamnoside 7-*O*-rhamnoside
**20**	42.9	577	431,285	Kaempferol-O-di-deoxyhexoside

Supplementary Table II. Antibodies used in the study

Antibodies	Providers	Number	Isotype	Dilution	Molecular weight (kDa)
**PARP**	Cell signaling	#9532	Rabbit	1:1000	116,89
**Procaspase3**	Cell signaling	#9661	Rabbit	1:1000	19,17
**Procaspase 7**	Cell signaling	#12827	Rabbit	1:1000	35,20
**Procaspase 8**	Cell signaling	#4927	Rabbit	1:1000	10,57
**Procaspase 9**	Cell signaling	#9508	Mouse	1:1000	49,37,35
**BOK**	Cell signaling	#4521	Rabbit	1:1000	22
**Bak**	Cell signaling	#12105	Rabbit	1:1000	25
**Bax**	Cell signaling	#14796	Rabbit	1:1000	20
**Bid**	Cell signaling	#2003	Rabbit	1:1000	22
**Bcl2**	Cell signaling	#3489	Rabbit	1:1000	26
**Bcl-xl**	Cell signaling	#2764	Rabbit	1:1000	30
**Smac/Diablo**	Cell signaling	#15801	Rabbit	1:1000	21
**Cytochrome c**	Cell signaling	#12963	Mouse	1:1000	14
**CoxIV**	Cell signaling	#11967	Rabbit	1:1000	17
**HSC70**	Cell signaling	#4872	Rabbit	1:1000	70
**β-actin**	Cell signaling	#3700	Rabbit	1:1000	42
**Anti-mouse IgG HRP**	Sigma Aldrich	71045-M	Goat	1 : 3000	
**Anti-rabbit IgG HRP**	Cell signaling	#7076	Goat	1 : 3000	

Supplementary Figure 2. ESI-MS/MS profiles of identified compounds

Caffeic acid derivative at [M - H]– 377

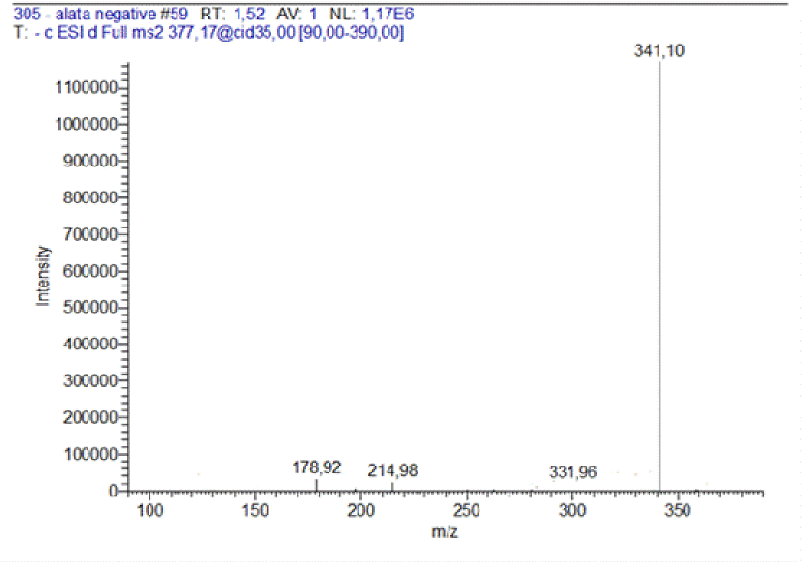

Citric acid at [M - H]– 191

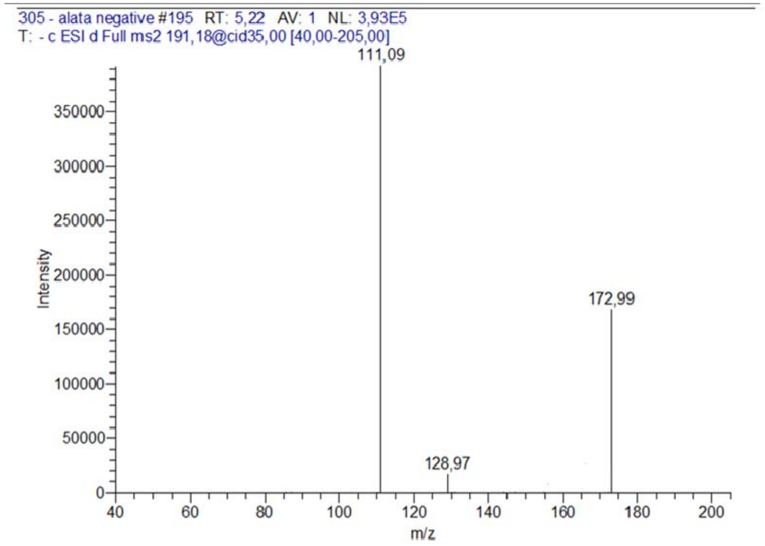

Syringic acid hexoside at [M - H]– 359

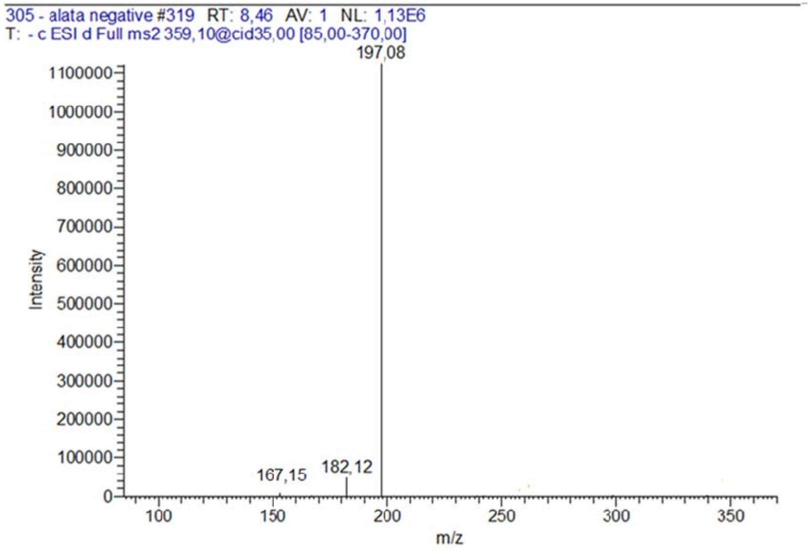

Syringic acid at [M - H]– 197

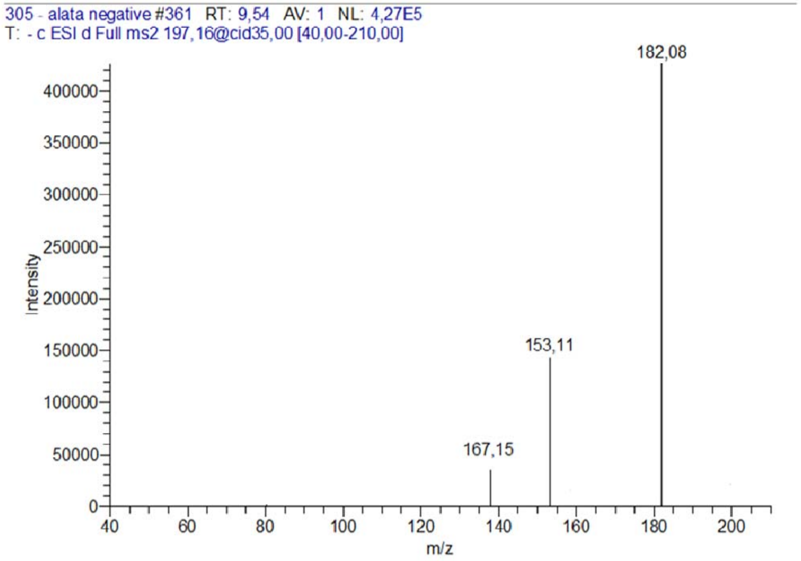

Supplementary Figure 2. ESI-MS/MS profiles of identified compounds-Continued

Gallocatechin at [M - H]– 305

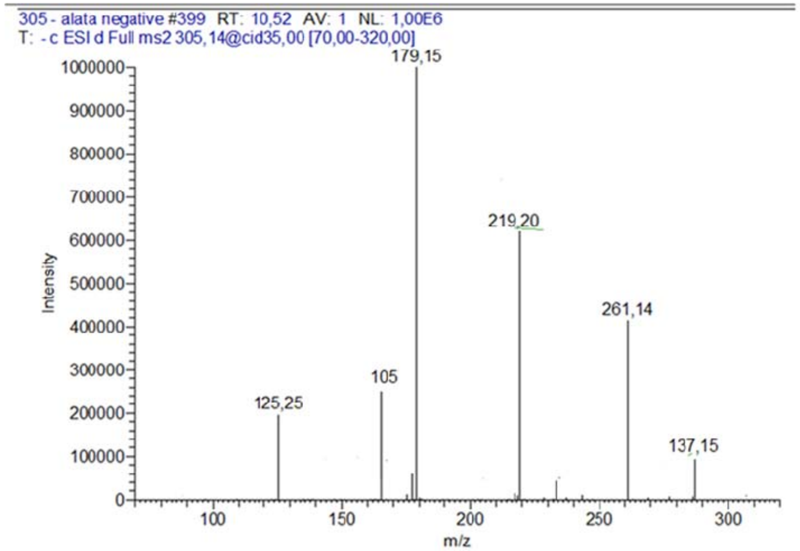

Ferulic acid at [M - H]– 193

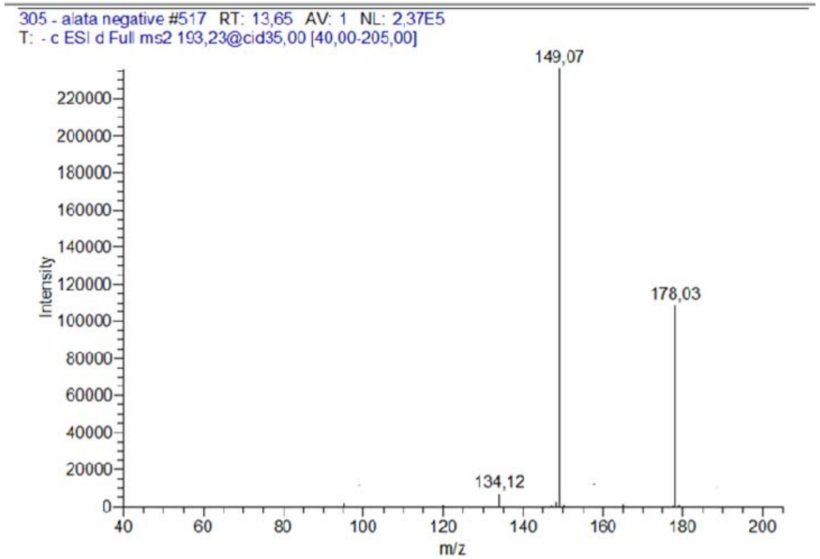

Quercetin dihexoside at [M - H]– 625

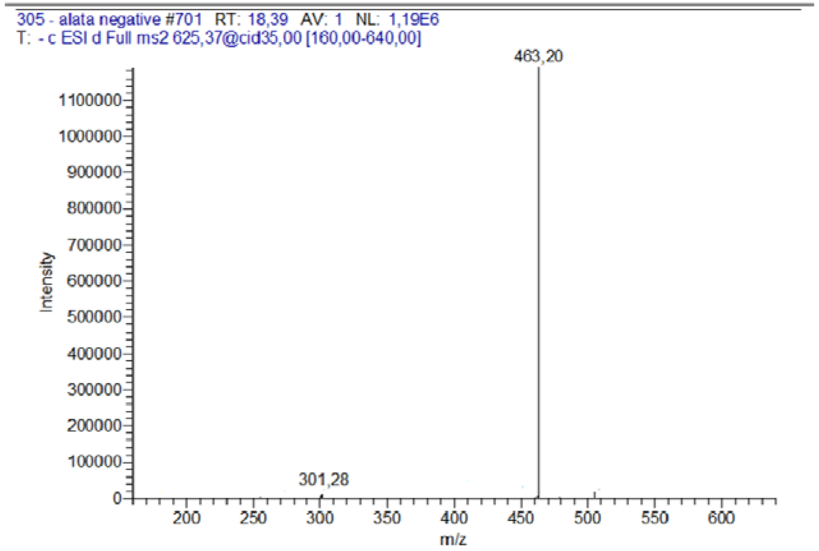

Vicenin-2 at [M - H]– 593

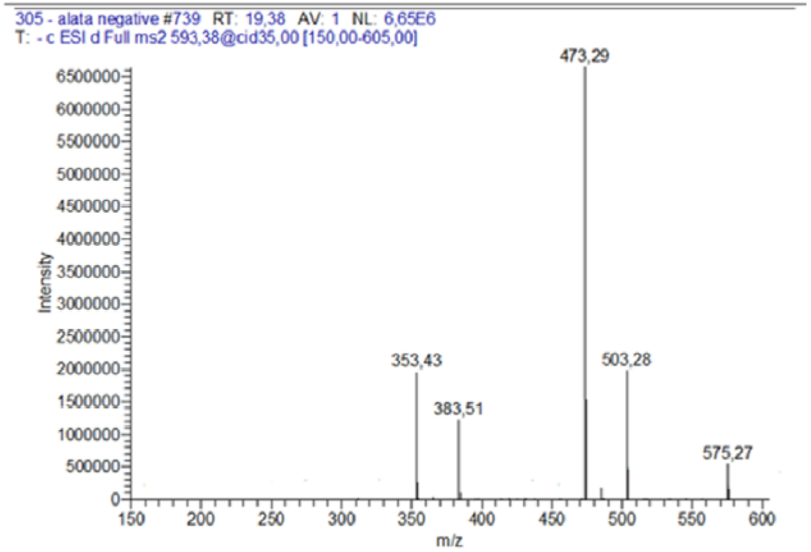

Supplementary Figure 2. ESI-MS/MS profiles of identified compounds-Continued

Quercetin-3-O-galactoside at [M - H]⁻ 463

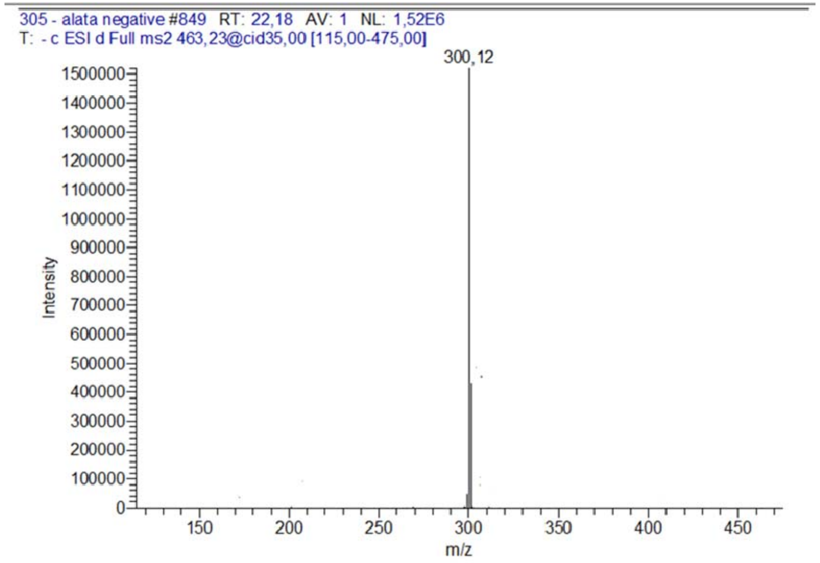

Isoorientin-4 -O-glucoside at [M - H]– 609

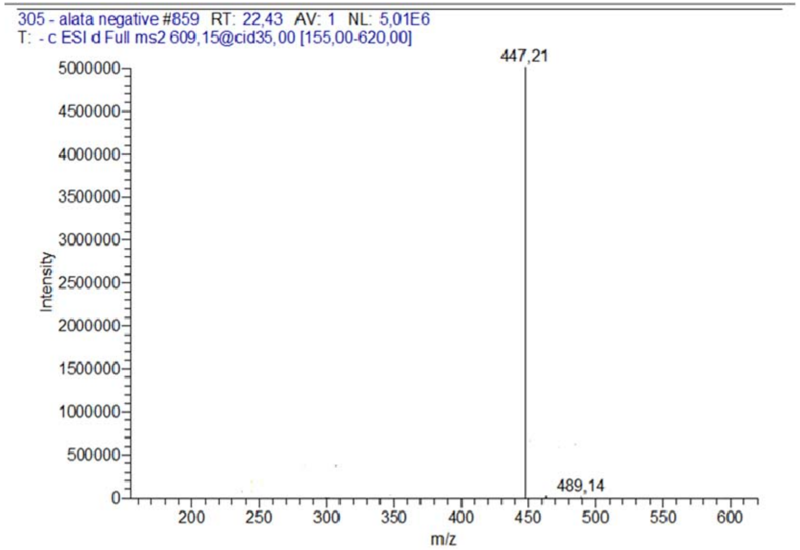

6-Hydroxyquercetin-3-O-di-hexose at [M - H]– 641

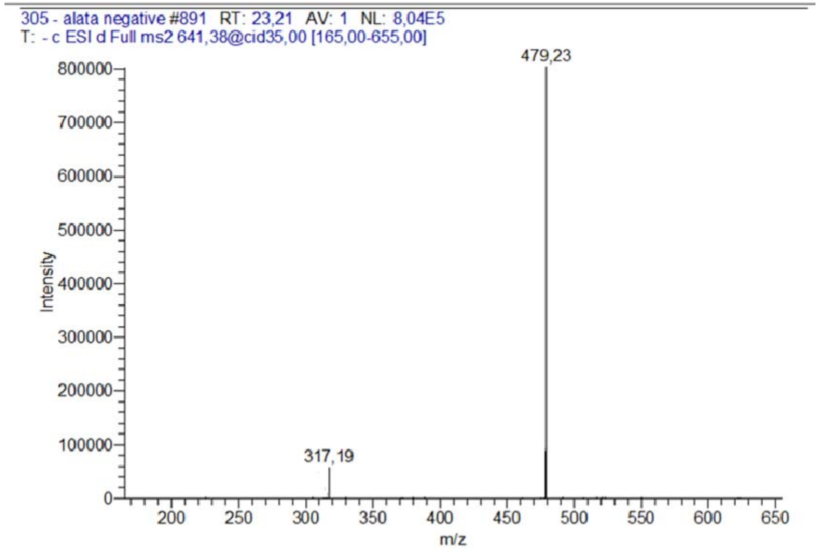

Quercetin 3-O-glucoside at [M - H]– 463

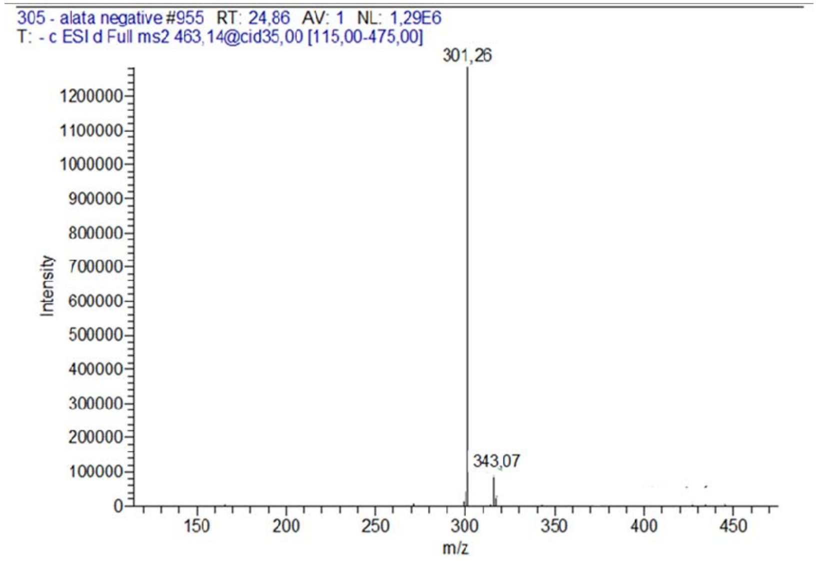

Supplementary Figure 2. ESI-MS/MS profiles of identified compounds-Continued

Luteolin-8-C-β-D-glucopyranoside at [M - H]– 447

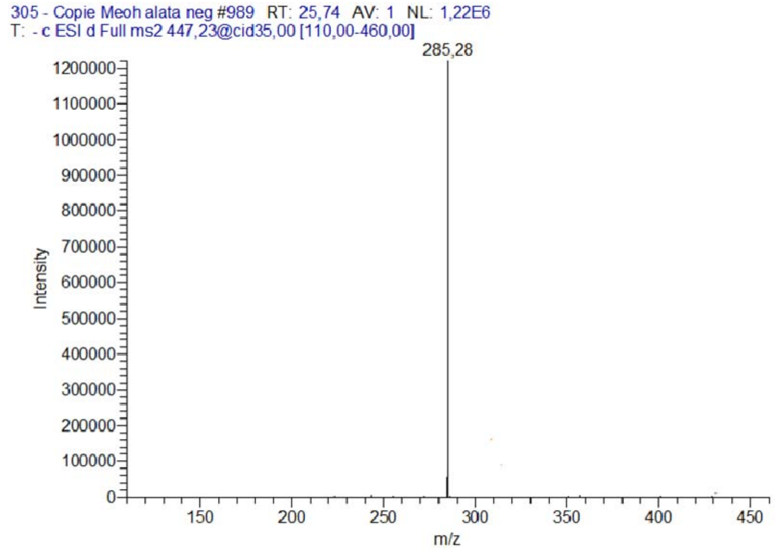

Quercetin at [M - H]– 301

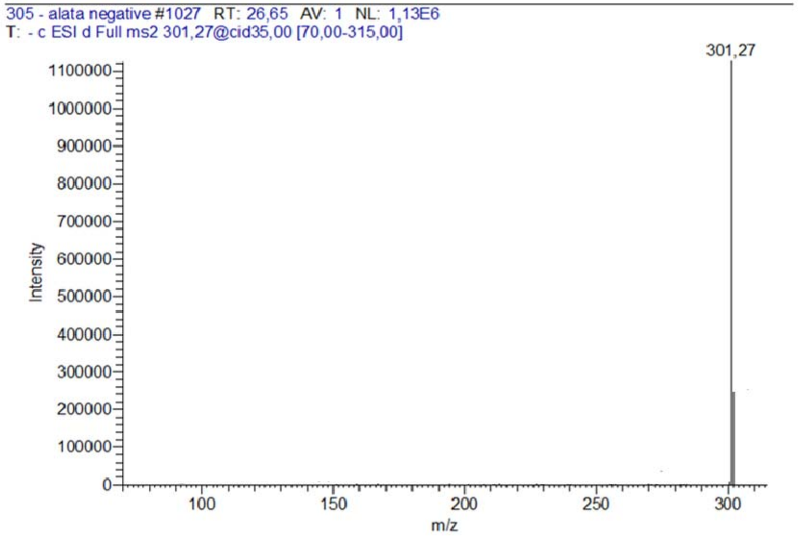

Myricetin-3-O-glucoside at [M - H]– 479

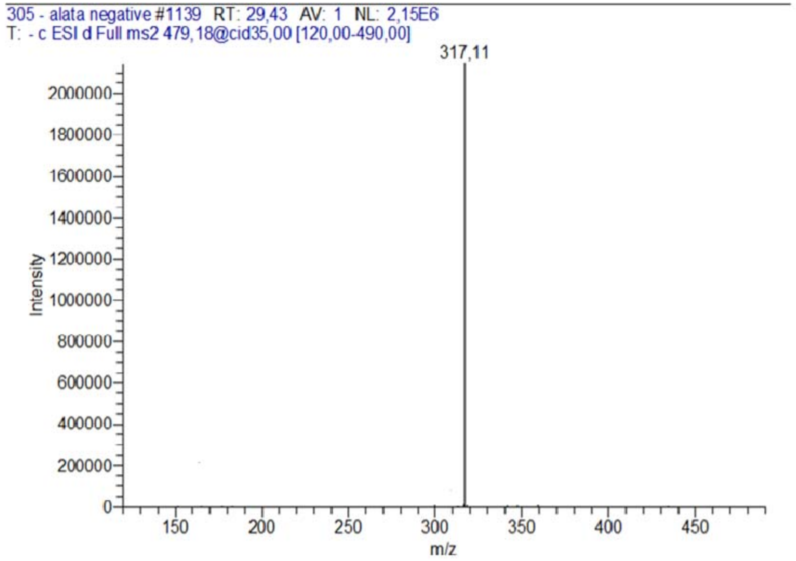

Quercetin 3-O-[6"-(3-hydroxyl-3-methylglutaryl)-β-D-galactoside] at [M - H]– 607

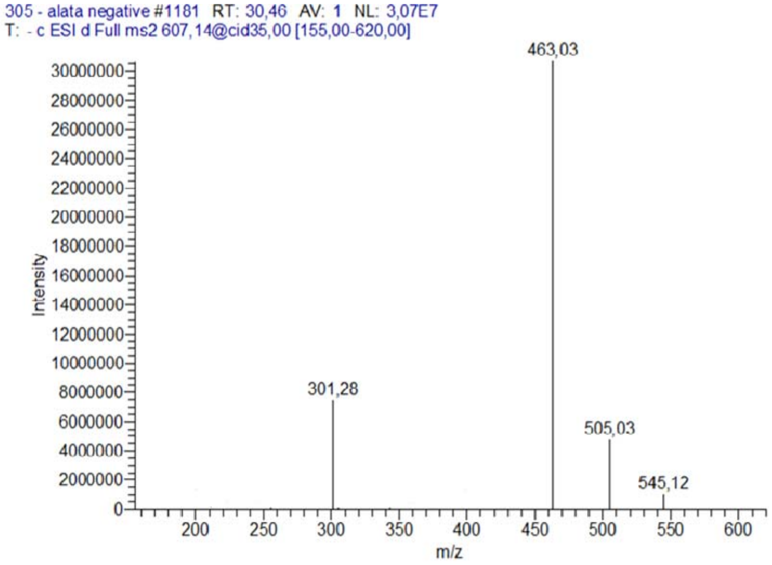

Supplementary Figure 2. ESI-MS/MS profiles of identified compounds-Continued

Quercetin 3-*O*-rhamnoside 7-*O*-rhamnoside at [M - H]– 479

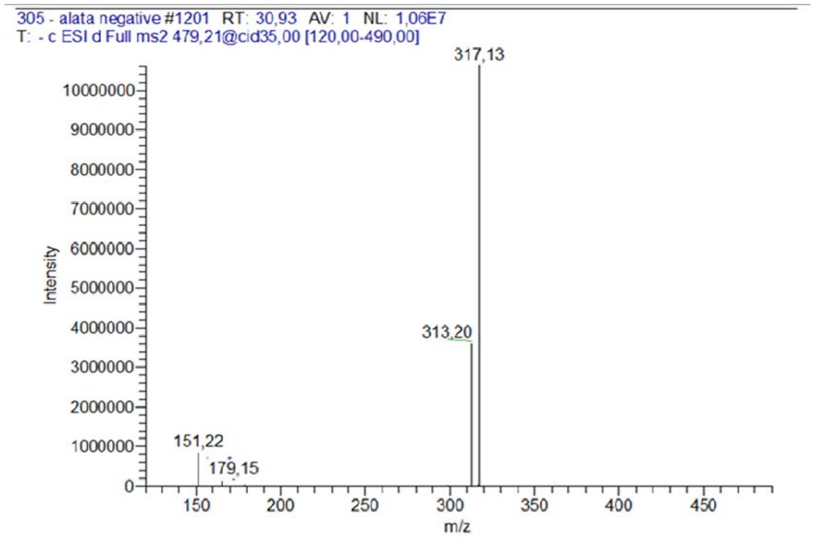

Unknown at [M - H]– 756

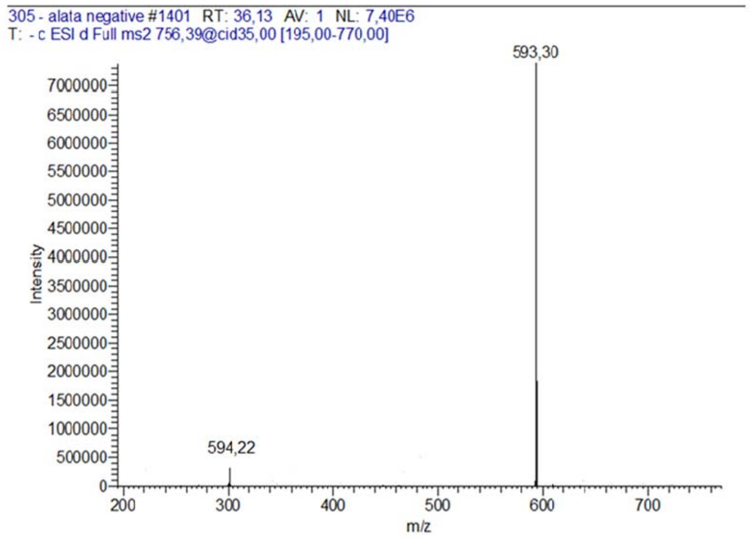

Quercetin 3-*O*-rhamnoside 7-*O*-rhamnoside at [M - H]– 593

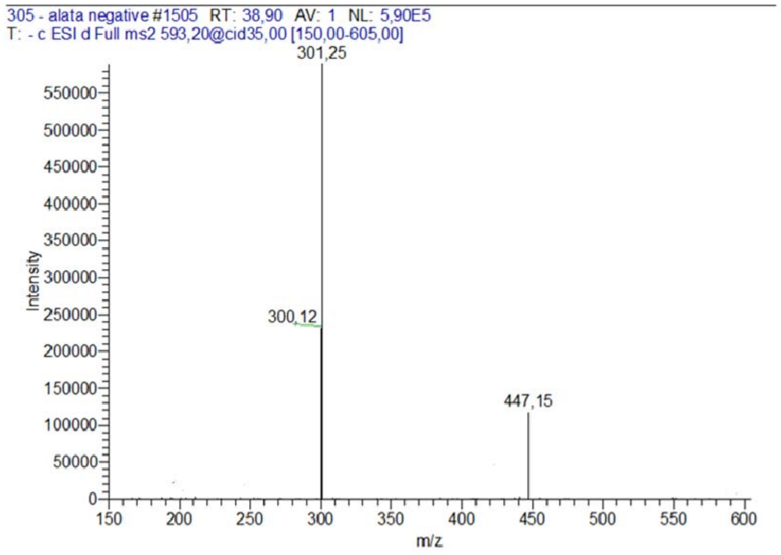

Kaempferol-O-di-deoxyhexoside at [M - H]– 577

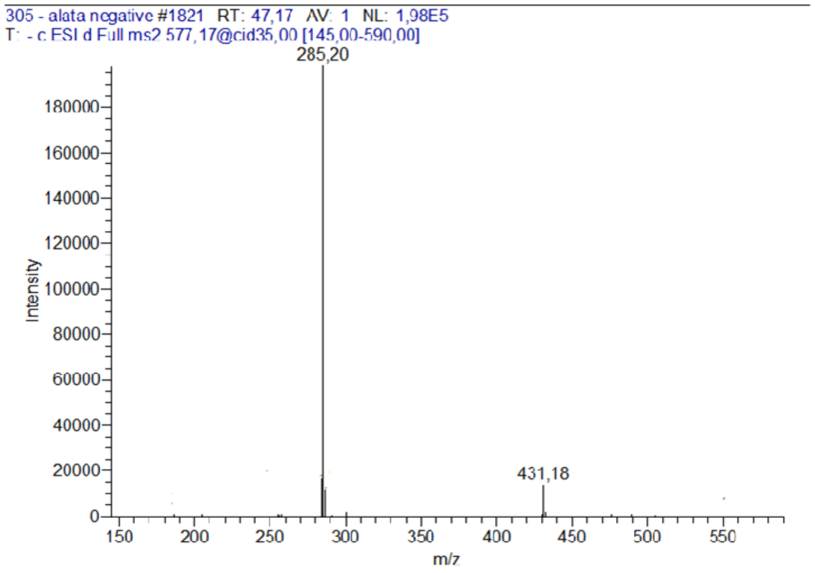

Supplement: Supplementary file 1 [file cells-09-00362-s001.pdf]
